# Control of morphology and crystallinity of CNTs in flame synthesis with one-dimensional reaction zone

**DOI:** 10.3762/bjnano.14.61

**Published:** 2023-06-21

**Authors:** Muhammad Hilmi Ibrahim, Norikhwan Hamzah, Mohd Zamri Mohd Yusop, Ni Luh Wulan Septiani, Mohd Fairus Mohd Yasin

**Affiliations:** 1 Department of Thermo-Fluids, Faculty of Mechanical Engineering, Universiti Teknologi Malaysia, 81310 Johor Bahru, Malaysia; 2 High Speed Reacting Flow Laboratory (HiREF), Universiti Teknologi Malaysia, 81310 Johor Bahru, Malaysia; 3 Advanced Membrane Technology Research Center, Universiti Teknologi Malaysia, 81310 Johor Bahru, Malaysia; 4 National Research and Innovation Agency, Indonesia

**Keywords:** carbon nanotubes, crystallinity, flame synthesis, morphology, one-dimensional flame, synthesis control

## Abstract

The growth of carbon nanotubes (CNTs) in a flame requires conditions that are difficult to achieve in a highly heterogeneous environment. Therefore, the analysis of the properties of the reaction zone within the flame is critical for the optimal growth of CNTs. In the present study, a comprehensive comparison between the CNT synthesis using a methane diffusion flame and a premixed flame is conducted regarding the morphology and crystallinity of the as-grown nanotubes. The premixed burner configuration created a flame that is stabilized through axisymmetric stagnation flow through sintered metal with one-dimensional geometry, different from a conventional co-flow flame. The significant difference in temperature distribution between the two flames causes a difference in the characteristics of the growth products. In the diffusion flame, the growth is limited to specific regions at certain height-above-burner (HAB) values with a temperature range of 750 to 950 °C at varying radial locations. The identified growth regions at different HAB values showed similar temperature distributions that yield CNTs of similar characteristics. Interestingly, the growth of CNTs in the premixed flame is dictated by only the HAB because the temperature distribution is relatively uniform along the radial directions but significantly different in the vertical direction. 17.3% variation in temperature in the axial direction successfully led to 44% and 66% variation in CNT diameter and crystallinity, respectively. The morphology control capability demonstrated in the present study is important for CNT functionalization for energy storage, nanosensor, and nanocomposite applications, where diameter and crystallinity are influential properties that govern the overall performance of the components.

## Introduction

Carbon nanotubes have been a research topic for a few decades since their discovery by Iijima in 1991 [1]. The CNT structure enables remarkable mechanical, electrical, and thermal properties. Studies of CNT syntheses using different methods yielding nanotubes with varying properties in terms of morphology, size, type, and functionalization have been reported [2]. Controlling the properties of nanotubes is essential for various applications. In a recent study on the production of CNT-based conductive textiles, the fabrication of conductive wool required the utilization of multi-walled CNTs (MWCNTs) with carboxyl functionalization [3].

The aerospace industry utilizes CNTs with high thermal, chemical, mechanical, and electrical performance as a nanocomposite additive to overcome problems in aircraft coatings, such as corrosion, ice accretion, lightning strike, and erosion. Overall, CNT/epoxy nanocomposites with superior mechanical properties are preferred [4]. Scarselli et al. [5] synthesized CNTs with a three-dimensional network using a sulfur enhancer and, thus, created a porous product capable of adsorbing oils. Furthermore, CNT networks have a high potential to be utilized in photon energy conversion devices due to the excellent photo response in the visible and near-UV light regions. Hence, controlling morphology and functionalization of such nanomaterials during synthesis will open vast opportunities to satisfy the requirements for various specific applications. To date, many researchers have shown promising results on the synthesis control of CNTs to produce tailored CNT morphologies and properties through conventional furnace-based methods.

Progress in CNT synthesis processes has been achieved mostly using chemical vapor deposition (CVD). Various studies on the growth control in CVD conclude that independent parameters, such as fuel selection, synthesis temperature, vapor pressure, and catalyst, govern CNT growth [6]; all can be manipulated for synthesis control. CVD is preferred because of the high degree of control and the possibility to synthesize at relatively low temperatures. However, CVD consumes a lot of energy for CNT growth, leading to increased production cost and, thus, making high-quality CNTs too expensive for practical applications. Alternatively, similar growth mechanisms of CNTs using the less common flame synthesis method are explored. In flame synthesis, the correct selection of the catalyst is the governing parameter to produce CNTs similar to those made in CVD in an economical production process [7].

Hamzah et al. [8] discussed the control of CNT morphology and growth in flame synthesis taking into account many parameters, such as fuel, catalyst, temperature, and diluent. However, the heterogeneous gas properties during combustion lead to a high temperature gradient and a wide distribution of chemical species within the flame. The interrelated processes of heat generation and carbon supply requires an optimum parametric control to achieve stable growth conditions. Yet, achieving independent parametric control in flame synthesis is a challenging task. In a diffusion flame, specific locations within the flame are imperative and conducive for CNT growth, depending on height above burner (HAB), local distance from the flame front, and flame composition [9].

A premixed flat flame was utilized by Zhang et al. [10] to synthesize single-walled CNTs (SWCNTs) through rich hydrogen/air combustion with a ferrocene catalyst. The temperature profile was estimated to be relatively uniform across HAB values up to 10 mm. An increase in CNT yield was observed at a high mass flux of hydrogen/air at a fixed equivalence ratio, resulting in higher temperature and an increase in *I*_G_/*I*_D_ ratio of up to ten times. A similar approach in spray pyrolysis CVD by Casanova et al. [11], utilizing ferrocene catalyst with cyclohexanol as a carbon source, showed that different growth temperatures produce CNTs with similar crystallinity. In a study by Chong et al. [12], the combustion of propane in a premixed flame at varying equivalence ratios yielded similar effects on CNT growth, while transmission electron microscopy (TEM) analysis showed a morphology of the MWCNTs similar to that of MWCNTs obtained from CVD. Raman spectroscopy showed constant *I*_G_/*I*_D_ ratios after all runs, and thermogravimetric analysis (TGA) results showed almost equal CNT oxidation temperatures, indicating similar purity. The constant crystallinity determined from the *I*_D_/*I*_G_ ratio was also shown in another study by Hamzah et al. [13], utilizing a methane diffusion flame with different air flow rates.

This study aims to synthesize CNTs on nickel wire through methane combustion in a one-dimensional premixed flat flame. The resulting CNT morphology and crystallinity will be compared and analyzed with CNTs grown in a methane diffusion flame. Ultimately, the use of a one-dimensional premixed methane flame, allows for a better understanding of the governing parameters for synthesis control.

## Results and Discussion

### Flame characterization and temperature

**Figure 1:**
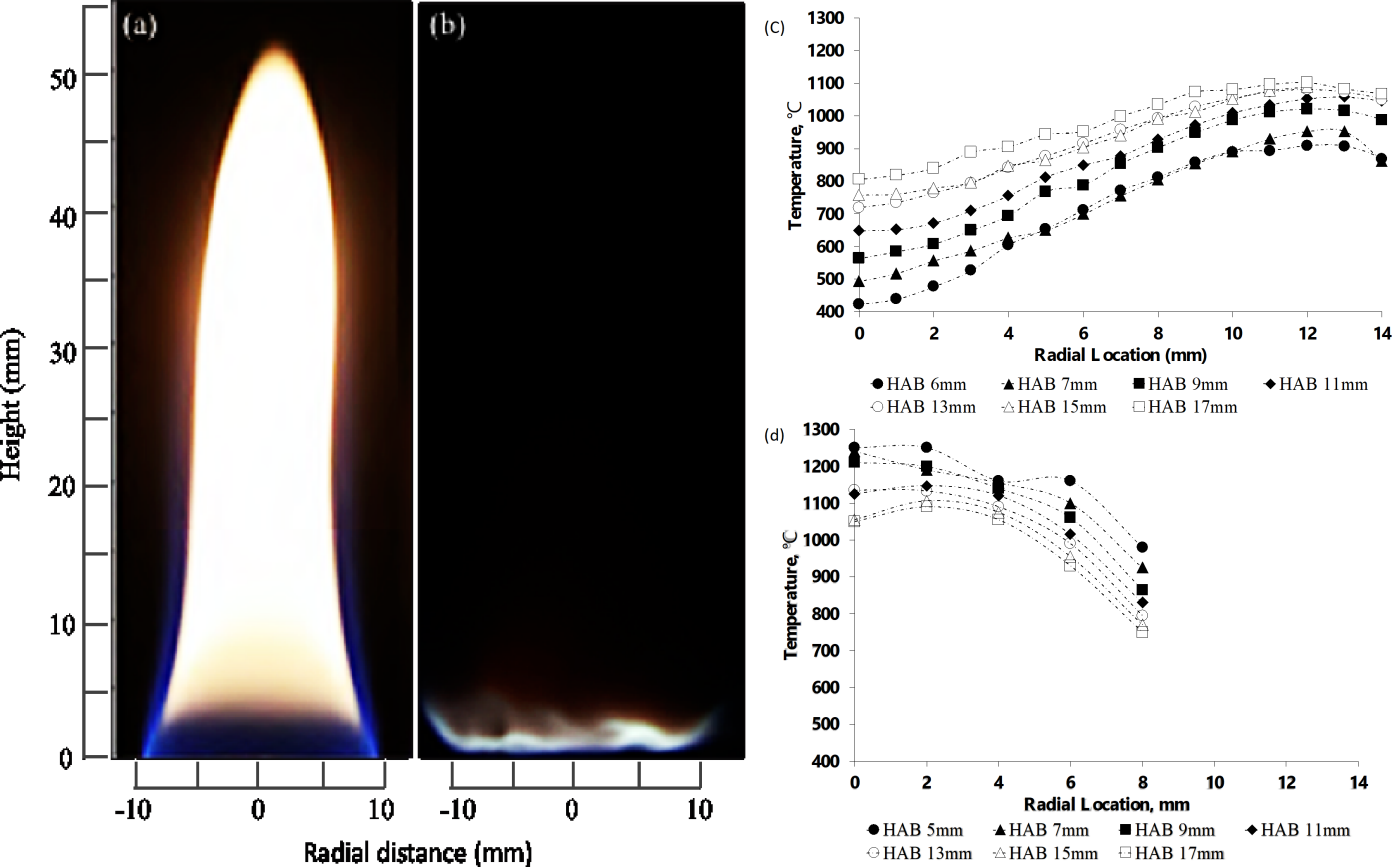
Line-of-sight images of standing flames and temperature distribution of (a,c) diffusion flame and (b,d) premixed flame. Figure 1a was used with permission of Elsevier Science & Technology Journals, from [9] (“Effect of fuel and oxygen concentration toward catalyst encapsulation in water-assisted flame synthesis of carbon nanotubes”, N. Hamzah et al., Combustion and Flame, vol. 220, © 2020); permission conveyed through Copyright Clearance Center, Inc. This content is not subjected to CC BY 4.0.

The flames employed in the present study are characterized regarding flame shape, spatial distribution of the reaction zone, and temperature distribution. Figure 1a,b compares line-of-sight images of the diffusion flame and the flat premixed flame burning at rich combustion with equivalence ratios of 1.16 and 1.8, respectively, where the equivalence ratio is calculated based on the inlet conditions. The diffusion flame has a bright yellow color due to soot formation with a height of 55 mm. Figure 1c shows that in the diffusion flame the temperature increases at high HAB values in radial locations near the flame sheet. As expected, the temperature is low near the burner because of burner heat loss, while the heat generation within the reaction zone of the blue flame sheet increases the local temperature. For CNT growth, the ideal temperatures are between 750 and 950 °C [14–15]. Hence, in the diffusion flame, the CNT growth regions are located at higher HAB values and near the flame center [13].

In a premixed flame setup, the use of sintered metal allows for a uniform gas flow distribution producing a flat flame shape, as shown in Figure 1b. The one-dimensional uniform blue flame suggests a uniform temperature distribution across the radial locations. The shield at the outer annulus provides encapsulation of the primary flame and avoids the entrainment of ambient air into the reactant stream, which might lead to the formation of a secondary diffusion flame. Figure 1d indicates temperature uniformity at all HAB values and up to 8 mm radial distance from the flame center, which is not observed in the diffusion flame. The maximum temperature is at 0–2 mm from the centerline at the lowest HAB of 5 mm. However, a temperature drop is observed in the far-field region away from the flame center line. The difference in temperature between the flame center and the flame sheet is due to the cold shielding gas flow at the outer annulus. This trend is observed at all HAB values, although the temperature at all locations is still within the required range for CNT growth. The height of the flame only reaches up to 5 mm, hence the term “flat”. The flame height varies according to the equivalence ratio and oxygen concentration of the flame [16]. A separate test showed that the flame loses its one-dimensional characteristics at an O_2_ concentration below 34%. A previous study showed that the consistency of the one-dimensional flame is vital to achieve uniformity of the flame characteristics in the radial direction [17], which is useful for consistent CNT growth along the metal wire in the present study.

### CNT growth

In general, the growth of CNTs on nickel wire starts from a process of surface breakup in a carbon-rich high-temperature environment, which forms a rough surface covered with nanoparticles. The carbide layer formed on the wire will induce localized stress on the surface due to lattice mismatch and, consequently, a breakup along grain boundaries to yield particles of different sizes and shapes [13]. According to the widely accepted vapor–liquid–solid mechanism, the growth of CNTs occurs in three steps, namely, melting of nickel particles, adsorption of carbon atoms onto the surface of the metallic nickel, and finally, diffusion and deposition of the precipitated carbon, which forms tubular materials by curling of graphene sheets on the surface [18–19]. In flame synthesis, the rapid heating rate causes catalytic activation and nucleation to occur almost instantaneously by the arrangement of carbon atoms on the surface of catalyst nanoparticles, leading to cap formation and liftoff.

**Figure 2:**
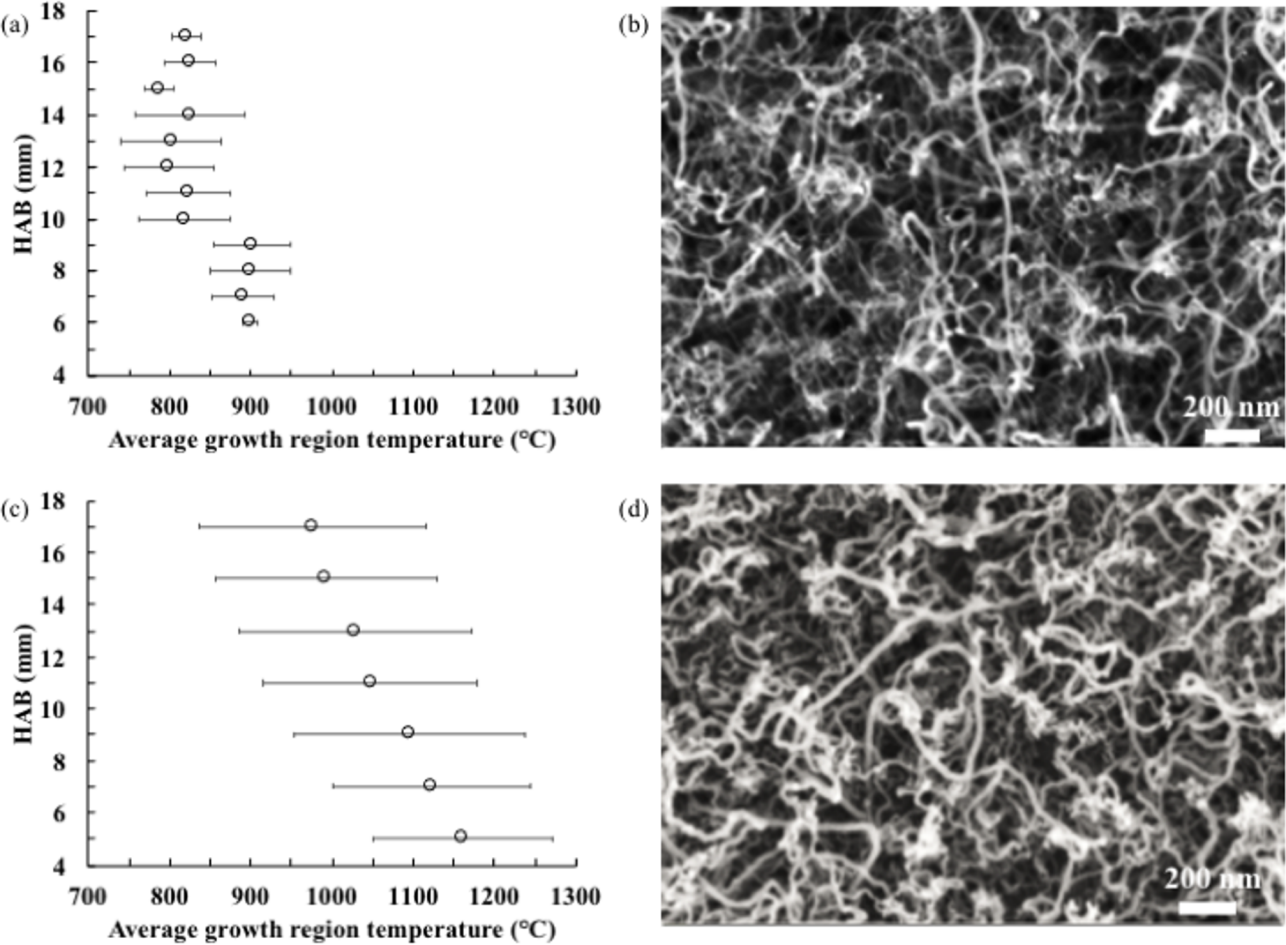
Average growth region temperature and the corresponding FESEM images of CNTs on nickel wire synthesized by (a,b) diffusion flame and (c,d) premixed flame.

Figure 2b and Figure 2d show FESEM images at 50.000× magnification of the CNTs grown on nickel wire in the diffusion and the premixed flame, respectively. The synthesized CNTs formed randomly oriented, long, curly and bent tubes with catalyst particles attached to the tubes. Generally, CNTs will follow a particular atomic arrangement and grow vertically from the substrate [20]. Said growth model is similar to the mechanism proposed by Baker and co-workers [21–23]. At elevated temperatures, catalytic particles are formed on the substrate surface, and hydrocarbon molecules that undergo cracking in the flame diffuse to the catalyst’s surface. Carbon atoms are adsorbed by the catalyst and deposited by diffusion to form nanotubes through continuous stacking. The weak interaction force between the catalyst particle and the substrate lifts the particles as the nanotubes grow, forming CNTs with catalyst particles at the tip. CNTs are formed with catalyst particles at the bottom if the catalyst–substrate interaction force is more substantial [23]. Nevertheless, the rapid growth of CNTs was observed from the catalytic reaction within the flame environment.

Because of the coupled energy and mass transfer phenomena, the temperature field and the gas phase chemistry evolve concurrently inside the flame [24]; thus, particle formation and CNT growth occur almost instantaneously. The rapid particle formation from the surface breakup produces a heterogenous particle size distribution. Consequently, particle bundles can be observed, revealing the agglomeration and sintering of particles during high-temperature growth in the flame. The energy dispersive X-ray (EDX) analysis results in Table 1 indicate a high fraction of nickel. A previous study showed that nickel particles of 5 nm and above can be reactive towards CH_4_, which is favorable for CNT growth [12].

**Table 1:** Average elemental composition of CNTs on nickel wire measured with EDX.

Element	C	O	Ni

content [wt %]	83.4	4.75	11.85
variance σ [wt. %]	2.50	0.85	2.13

The average growth temperatures for diffusion and premixed flames are shown in Figure 2a and 2c, respectively. In the diffusion flame, CNT growth extends from the close vicinity of the flame sheet towards the flame centerline, within the fuel stream with a high concentration of carbon sources. The growth regions end where the local temperature falls below the minimum growth temperature. The growth temperature has been measured at the growth regions. There is a difference of average temperature of about 100 °C between growth regions below 9 mm and above 10 mm. The temperature of the high-HAB region (above 10 mm) is lower because of the relatively faster carbon formation process due to the abundance of carbon sources and the adequate temperature, specifically within the fuel stream flow. Contrarily, in the low-HAB region (below 9 mm), there is no growth within the fuel stream because of the lower temperatures. Instead, growth occurs inside the oxidizer stream flow at the flame sheet, although the growth region shrinks because of a lack of carbon sources.

In the premixed flame, the temperature across the radial locations is relatively uniform; thus, CNT growth can occur radially in all regions. The temperature uniformity across the radial locations is expected to produce less heterogeneous particle sizes. The average growth temperature in the premixed flame has been measured for all regions. Interestingly, there is a significant difference of 200 °C between the temperatures at lower and higher HAB values. The lowest HAB value shows the highest temperature reading, which linearly decreases with increasing HAB values. The temperature differences are expected to produce different particle size distributions due to aggregation of particles on the surface of the nickel wire. The CNT diameters, which often correlate to the particle size, show a similar trend, as discussed in the next section.

### CNT characterization

**Figure 3:**
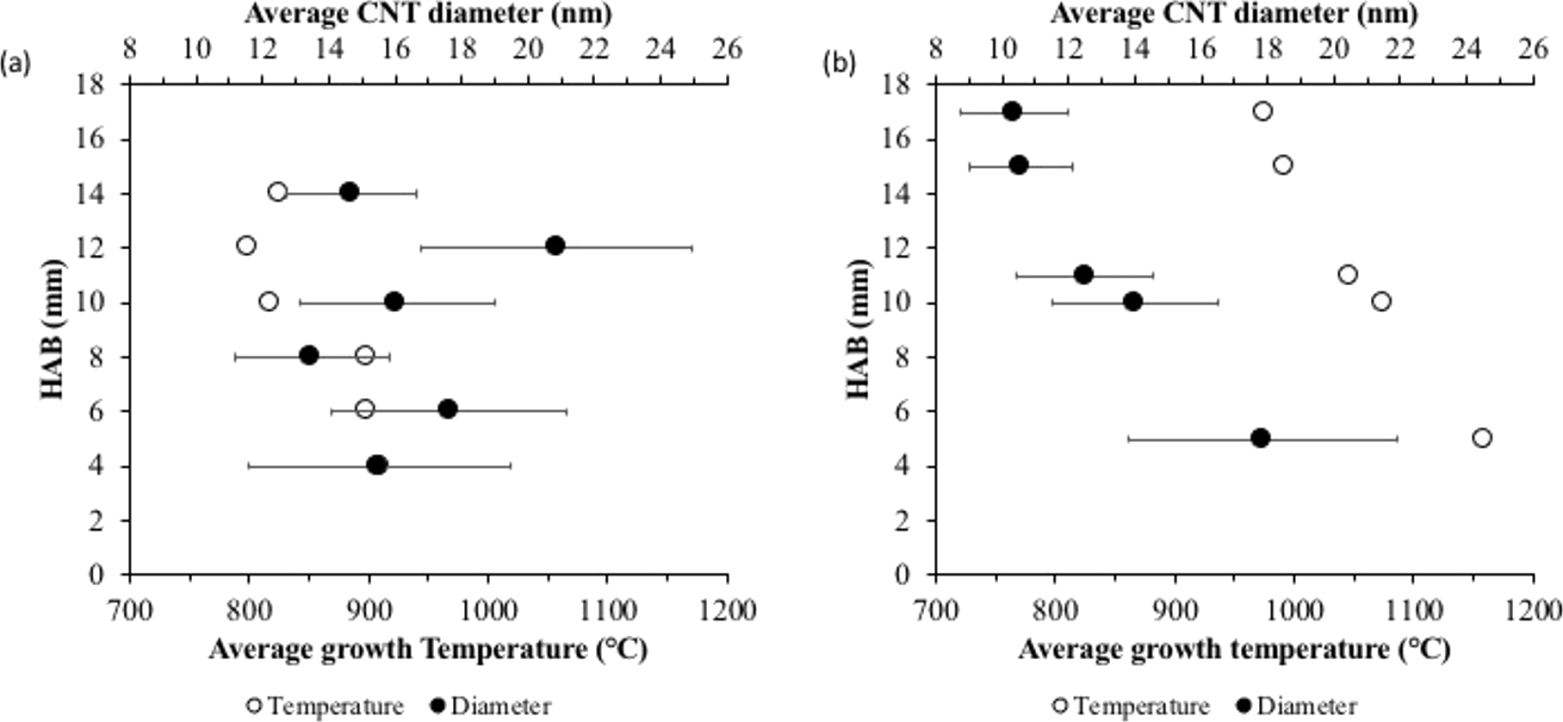
Average CNT diameter distribution and average growth temperature for (a) diffusion flame and (b) premixed flame.

The diameter of the synthesized CNTs has been measured using the image post-processing software Digimizer^TM^ with 200–300 data points per sample to produce the size histograms. Figure 3a and Figure 3b show the temperatures and CNT diameter distributions for methane diffusion flame and premixed flame, respectively. For the diffusion flame synthesis, a clear gap in the average temperature of around 100 °C is observed for the CNT growth regions below and above 10 mm HAB. The average temperature for growth regions below 10 mm HAB falls within the narrow range of 880 to 910 °C, whereas for regions above 10 mm HAB, the average growth temperature is between 790 to 830 °C. As reported by Hamzah et al. [9], at low HAB values, the growth region is located in a high-temperature region close to the flame sheet and the border between fuel and oxidizer streams of the burner. Whereas, at higher HAB, the growth region is concentrated at the flame centerline. However, despite the difference in temperature, the morphology and characteristics of the CNTs synthesized in these regions are not significantly different. The CNT diameter distributions at every sampled HAB value show no significant trend, with an average diameter ranging from 13–21 nm. The standard deviations indicate a large diameter variation. Nevertheless, the consistency in CNT diameter at all HAB values shows stable growth regions that yield CNTs of the same morphology. The identified regions are likely to share a common distribution of temperature and precursor concentration (scalar) that is favorable for the formation of CNTs. Although the scalar distribution could vary the formation rate, there is no effect on the size of the formed CNTs.

Figure 3b shows a linear trend in the CNT diameter distribution at different HAB values of the premixed flat flame. Small average CNT diameters were obtained at high HAB values with a maximum variation of 10 nm in diameter. At the low HAB value, the large average CNT diameter with high standard deviation suggests different sizes of formed particles, possibly due to the high temperature. Generally, the size of nanoparticles is affected by several factors, including solution concentration, deposition method, quantity, and annealing [25]. At higher temperatures and lower HAB values, the particles can combine, sinter, and increase in size either due to Ostwald ripening or particle migration and coalescence [26]. The agglomeration occurs because of the high temperature, creating conditions similar to annealing. At higher HAB values, where the temperature is relatively low, the average CNT diameters decrease with smaller standard deviations, implying the formation of more uniform particle sizes. Overall, the flat premixed flame yielded 17% of temperature variation in the axial direction, resulting in 44% of CNT diameter variation.

**Figure 4:**
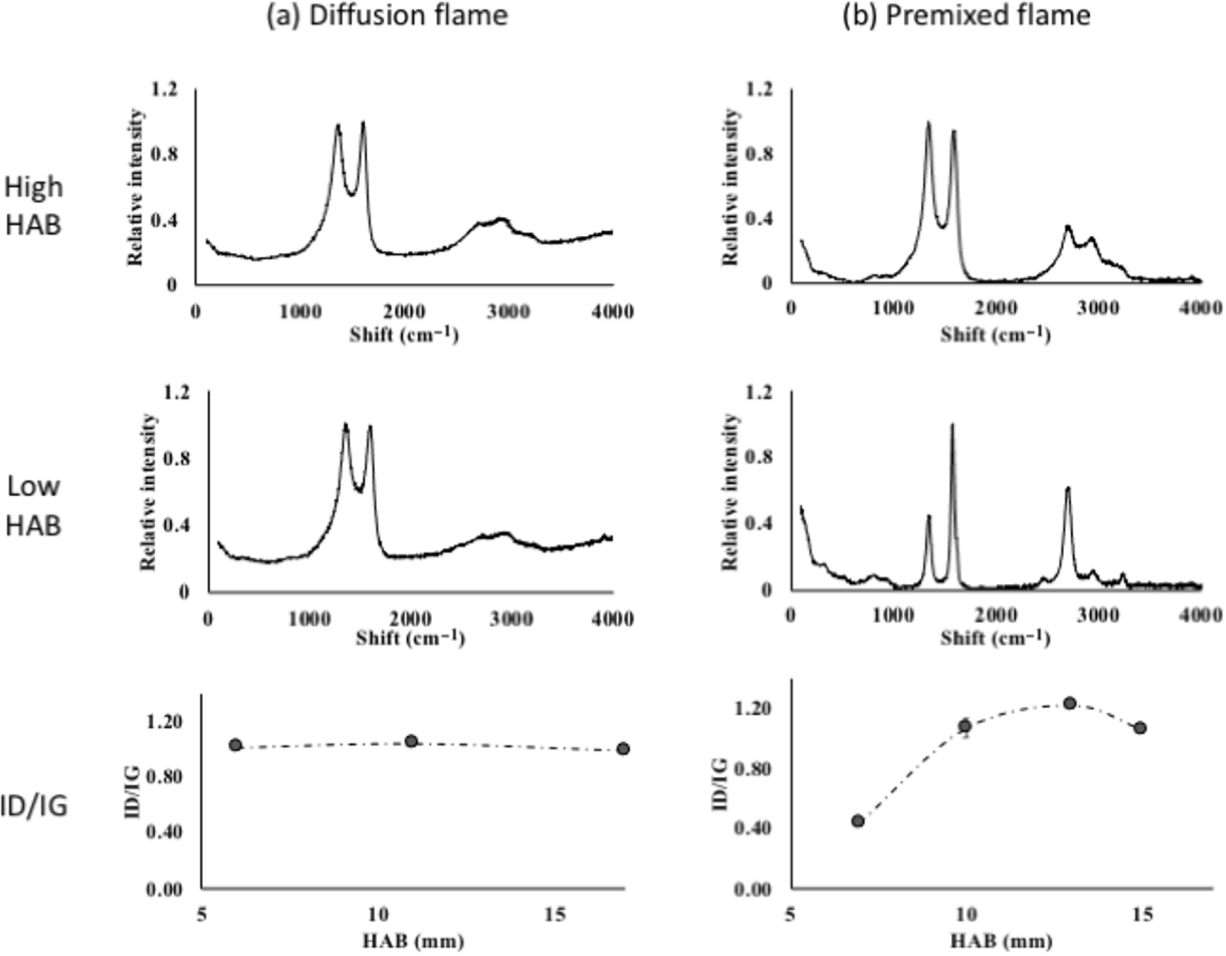
Raman shifts of CNTs at low, high, and average HAB values for (a) diffusion and (b) premixed flame.

Figure 4 shows the Raman spectra of the CNTs grown in diffusion flame and premixed flame at the highest and the lowest HAB values. Generally, the formation of sp^2^-hybrdized carbon atoms is often correlated to Raman spectra having G peaks at 1550–1600 cm^−1^. Similarly, a D peak around 1250–1450 cm^−1^ often correlates to defects and disorders of the sp^2^-hybridized sidewalls, while the G′ peak at 2500–2900 cm^−1^ represents photon–phonon interactions [27].

The absence of a low-frequency peak below 200 cm^−1^, usually assigned to the A_1g_ symmetry radial breathing mode, which is the main characteristic of SWCNTs [28], indicates that the products of both syntheses are MWCNTs. In addition, the broad asymmetric feature in the G band is a property of MWCNTs, rather than of SWCNTs or graphite, which have a double peak or a narrow peak, respectively. The D band (*I*_D_) and G band (*I*_G_) intensities show an *I*_D_/*I*_G_ ratio of less than 1.00, indicating high crystallinity and purity of the CNTs. DiLeo theorized that the purity can be determined from the *I*_G’_/*I*_D_ ratio, where a higher ratio indicates a higher purity [29].

In the diffusion flame, the *I*_D_/*I*_G_ ratios at low, middle, and high HAB values are almost constant within the range of 0.98 to 1.01, indicating consistency in crystallinity. The almost identical spectra of the CNTs at all HAB values indicate uniformity in terms of particle formation and CNT growth, as shown by the diameter distribution. As the identified growth regions create similar growth conditions, the process at every HAB value yields CNTs of similar purity and crystallinity.

The *I*_D_/*I*_G_ ratio increases at high HAB values in the premixed flame. As expected, the carbon supply at low HAB values is higher, leading to a higher graphitic band peak. The increase in D band peaks with low MWCNT content at high HAB values directly results from carbonaceous by-products. However, the *I*_D_/*I*_G_ ratio seems to settle at around 1.00 at a much higher HAB value. Hence, crystallinity and graphitization of the products are inversely proportional to the diameter, as discussed previously, leaving a trade-off that requires optimization. Overall, the 17% temperature variation has yielded 66% variation in crystallinity of the grown CNTs.

## Conclusion

Controlling the morphology of CNTs through flame synthesis remains a challenging topic, although it is highly useful for the functionalization of nanomaterials for specific applications. The heterogeneous temperature distribution within flame structures yields particles of varying sizes and shapes. Using a one-dimensional flame is expected to increase the controllability of synthesis parameters and to yield more uniform CNT properties. This study explores CNT growth on nickel wire using a one-dimensional premixed flat flame and compares the resulting temperature distributions and CNT morphologies and crystallinities with that of diffusion flame growth. The highly non-uniform temperature distribution of the diffusion flame allows for CNT growth only in specific locations within the flame, resulting in similar characteristics of the as-grown products. In contrast, the premixed flame successfully exhibits a uniform temperature distribution across the radial direction with a 17% variation in temperature observed only along the axial direction between the lowest and highest HAB values. The resulting CNTs show a linear trend in diameter with a 44% variation between the lowest and highest HAB values and a 66% variation in crystallinity. The demonstrated control of CNT diameter and crystallinity based on the HAB is a unique capability of the present one-dimensional flame configuration, which might lead to improved flame synthesis systems in the future.

## Experimental

### Catalyst preparation

**Figure 5:**
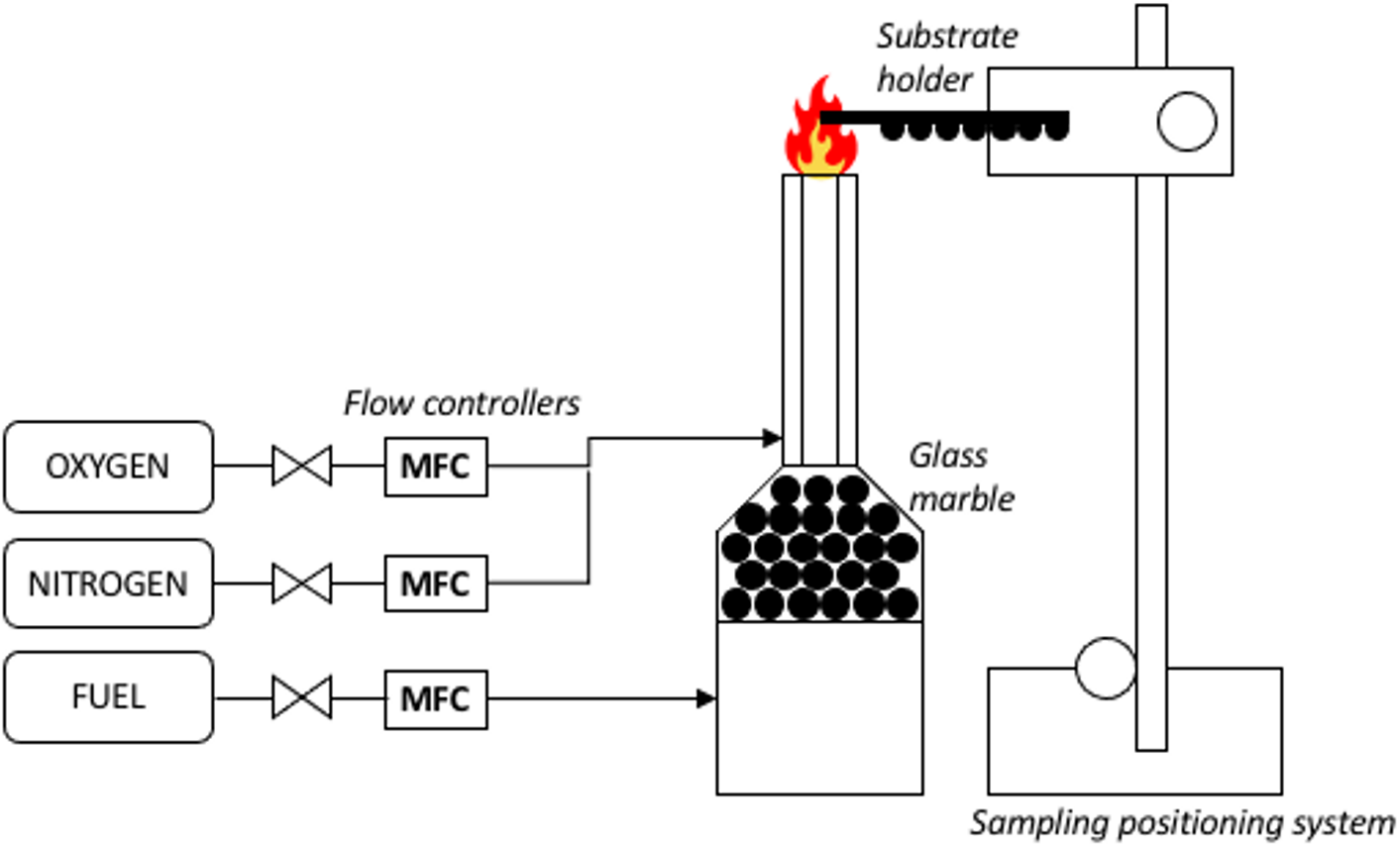
Laminar diffusion flame synthesis setup employing a traversing arm with an accuracy of 1 mm.

In the context of surface breakup catalysis, nickel wire is utilized as a catalyst through the formation of nickel nanoparticles. The method for preparing the nickel wire has been previously documented in [13]. The process involves cutting a 0.4 mm diameter nickel wire (TEMCO Inc.) into pieces of 50 mm length, followed by cleaning through sonication in an acetone solution for 15 min, and subsequently in distilled water. The wires are then air-dried before being inserted into a flame using a substrate holder (see below in Figure 5).

### Diffusion flame setup and synthesis

The setup for diffusion flame synthesis, as depicted in Figure 5, involves the use of a laminar diffusion flame burner, with methane gas and oxidizer being fed through concentric outlet tubes of 17 and 26 mm diameter, respectively. The central tube supplies 99.995% methane at a fixed rate of 0.4 slpm, controlled by an Omron^TM^ flow sensor. The outer tube supplies the oxidizer gases, a mixture of 99.9% oxygen at a fixed rate of 0.7 slpm and 99.9% nitrogen at a fixed rate of 3.0 slpm, controlled by metering valves equipped with Honeywell^TM^ sensors. The flame is established with a global equivalence ratio of 1.16 and an oxygen concentration of 19%. The cleaned nickel wire is exposed to the flame using a traversing system, which positions the wire at desired location with an accuracy of 1 mm. Analogous to the previous publication [13], the synthesis starts with the nickel wire being exposed to an open flame at HAB values of 4 mm up to 14 mm for 3 min. The methane diffusion flame is unable to form under the wire mesh below 4 mm HAB, whereas carbon nanotubes (CNTs) are not synthesized on the nickel wire above 15 mm HAB. The entire synthesis is conducted within a burner enclosure at atmospheric pressure. An extractor fan is also employed to minimize surrounding air disturbance to the flame.

### Premixed flame setup and synthesis

**Figure 6:**
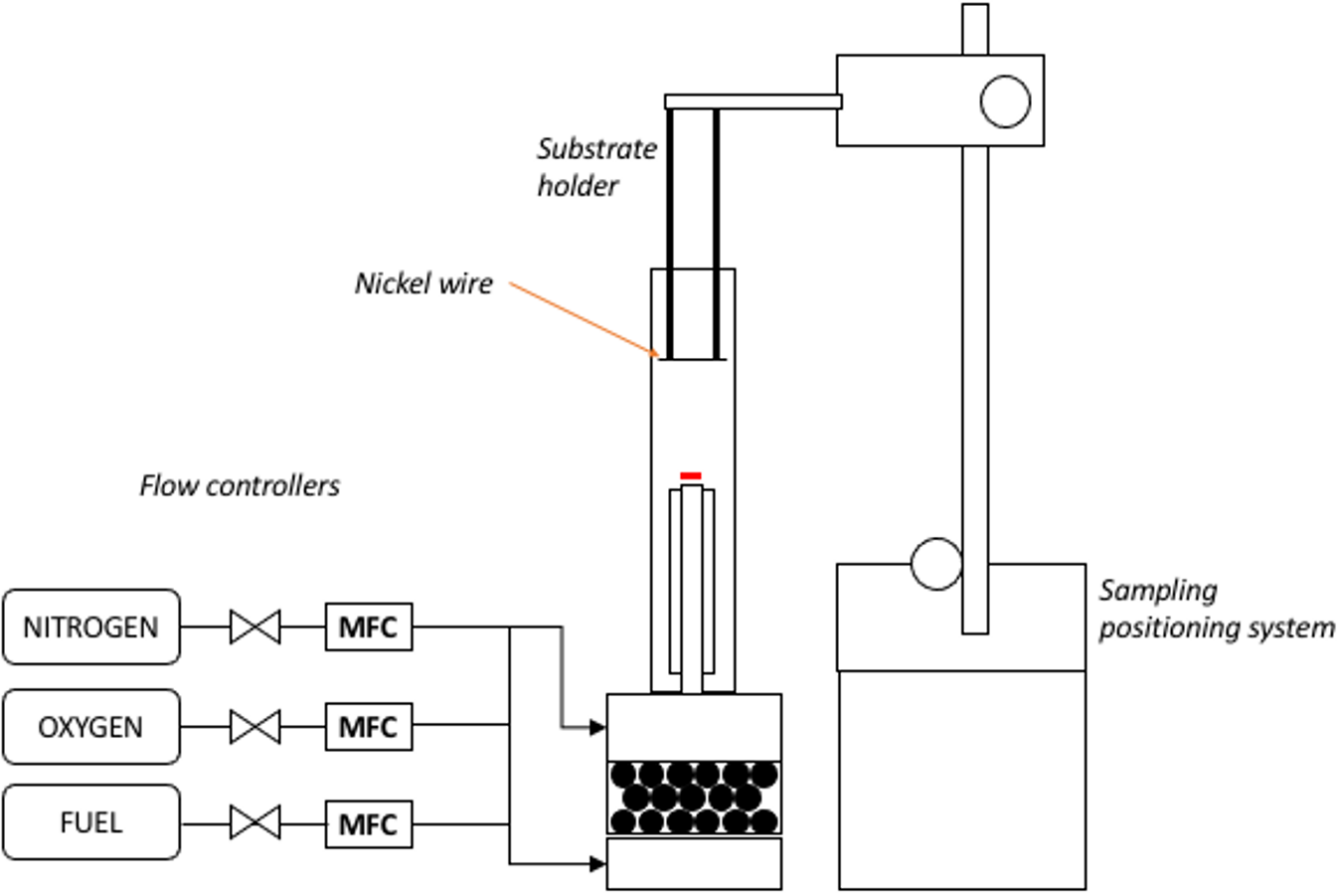
Schematic diagram of the premixed flame synthesis setup with quartz tube and traversing arm. The one-dimensional flame is achieved by the utilization of sintered metal at the outlet of the burner.

The setup for CNT synthesis using a premixed flame burner with a sintered metal outlet is depicted in Figure 6. The burner comprises a premix chamber and a concentric nozzle tube outlet with diameters of 17 and 26 mm, respectively. A quartz tube with an outer diameter of 50 mm is placed around the burner nozzle. To synthesize the CNTs, a positioning system with an accuracy of 0.5 mm is employed, and the sample is placed on top of a mesh wire attached at the bottom of the sampling probe. The probe is vertically inserted into the quartz tube from the top, and the HAB values are varied between 5 and 17 mm by adjusting the insertion length. The sampling time is fixed at 1 min for all runs unless specified otherwise.

The combustion of the premixed flame is achieved by mixing methane (99.995% purity), oxygen (99.9% purity), and nitrogen (99.9% purity), all controlled with Kofloc flow controllers at 0.4 slpm, 0.445 slpm, and 0.8 slpm, respectively. The mixture produces a flat flame burned at a 1.8 equivalence ratio and 34% oxygen concentration, stabilized by flowing nitrogen as a shield gas at 6 slpm, which simultaneously suppresses the secondary flame.

### Flame and CNT characterization

The flame temperature is measured using a type-B thermocouple with 0.5 mm bead size (Omega Engineering) and a traversing system with an accuracy of 0.5 mm. The thermocouple is mounted at the end of solid arms, and the traversing system moves the thermocouple bead to a specific location within the flame. For accuracy, measurements were done at different locations by moving the arm in and out of the flame.

The grown CNTs were characterized by field-emission scanning electron microscopy (FESEM, Zeiss Crossbeam 340) coupled with energy-dispersive X-ray analysis (EDX) for morphology and elemental analysis. Raman spectroscopy (HORIBA XploRA PLUS, 532 nm) was done to analyze the signature spectra of the grown CNTs.
